# Soft ground micro TBM jack speed and torque prediction using machine learning models through operator data and micro TBM-log data synchronization

**DOI:** 10.1038/s41598-024-60681-8

**Published:** 2024-04-27

**Authors:** Kursat Kilic, Owada Narihiro, Hajime Ikeda, Tsuyoshi Adachi, Youhei Kawamura

**Affiliations:** 1 Department of Geosciences, Geotechnology and Materials Engineering for Resources, Graduate School of International Resource Sciences, Akita University, Akita, 010-8502 Japan; 2 Department of Systems, Control and Information Engineering, National Institute of Technology, Asahikawa College, 2-2-1-6 Syunkodai, Asahikawa, 071-8142 Japan; 3 Division of Sustainable Resources Engineering, Faculty of Engineering, Hokkaido University, Kita 13, Nishi 8, Kita-Ku, Sapporo, 060-8628 Japan

**Keywords:** Optuna, Machine learning, Micro slurry TBM, Soft ground tunnelling, TBM jack speed control, TBM torque control, Operational parameters, Structural geology, Civil engineering

## Abstract

Tunnel Boring Machines (TBMs) are pivotal in underground projects like subways, highways, and water supply tunnels. Predicting and monitoring jack speed and torque is crucial for optimizing TBM excavation efficiency. Conventionally, skilled operators manually adjust numerous tunnelling parameters to regulate the machine's progress. In contrast, machine learning (ML) algorithms offer a promising avenue where computers learn from operator actions to establish parameter relationships autonomously. This study introduces an innovative approach to enhancing operator monitoring and TBM data comprehension. A robust correlation between TBM operator behaviour and TBM logged data is established by leveraging an Optuna-assisted ML methodology—the research light on the intricate dynamics influencing TBM advance rate parameters. Operational data is collected from micro slurry tunnel boring machine (MSTBM) umbrella support excavations. The proposed framework harnesses Optuna, an advanced hyperparameter optimization platform, to dynamically refine jack speed and torque settings. Through meticulous analysis of the interplay between TBM operator decisions and real-time logged data, the AI model discerns patterns, empowering informed decision-making. Using Optuna, a range of models, including random forest (RF), K-nearest neighbours (kNN), decision tree (DT), XGBoost, Support Vector Machine (SVM), and Artificial Neural Network (ANN) were automatically compared and tuned. The best model's (RF) performance is evaluated through a correlation coefficient (R^2^) of 96%, mean squared error (MSE) of 119.7, and mean absolute error (MAE) of 4.42 for jack speed decision making while 83% of R^2^, MSE of 0.62, and MAE of 0.42 for the torque decision making. This intelligent model can assist the TBM operator in making decisions about TBM control.

## Introduction

A tunnel boring machine (TBM) is an advanced engineering machine designed for automated tunnel excavation. Featuring a rotating cutter head and efficient cutting tools, it can effectively penetrate diverse geological formations, from soft soil and clay to resilient rock and abrasive materials. Employing cutting-edge technology and engineering principles enables TBMs to navigate even the most formidable terrains with precision and heightened efficiency. In contrast to traditional tunnelling methods, which often rely on extensive manual labour and heavy excavation machinery, mechanized tunnelling utilizing TBMs diminishes the necessity for human intervention at the tunnel face. This manual labour reduction significantly mitigates potential risks and enhances worker safety^1–3^.

Nevertheless, these benefits are relatively based on the experience of the TBM operator who monitors the TBM, as numerous operational parameters should be set for perfect control of the tunnelling operations. The mismatch between the TBM operator and TBM operational parameters results in low penetration efficiency, cutter tools wearing, and main bearings problems with machine jamming^4^. In this regard, the TBM cutter torque and jack speeds are essential to monitor the TBM advancement. They presented that the cutter-head torque lies in supplying the necessary cutting force for rock fragmentation during tunnelling. The cutter-head torque significantly impacts rock fragmentation efficiency and the interaction between the rock and the machine. The optimization of cutter-head torque, aimed at ensuring torque stability, has demonstrated favourable outcomes in terms of cutter-head load management and motor function. The optimization, in turn, facilitates enhanced construction speed, reduced mechanical losses, and lowered construction expenses. Accurately predicting cutter-head torque prevents entrapment and guides timely adjustments to TBM advancing parameters. In addition to the torque monitoring, controlling the jack speed is a significant operational parameter due to avoiding potential ground disturbance and maintaining the face stability of the tunnel during excavation^5^. Under these circumstances, the TBM torque and jack speed prediction have been researched into three main groups: empirical, probabilistic, and artificial intelligence models. The empirical models are based on laboratory scale tests such as the Colorado School of Mines (CSM) model^6,7^, the Norwegian University of Science and Technology (NTNU) model^8^, the rock mass rating (RMR) system^9^, and the QTBM models^10^. Nevertheless, these models do not account for variations in tunnelling conditions or do not consider operator decisions on TBM monitoring and focus only on a limited set of parameters^11–13^. The traditional probabilistic models have been proposed^14–18^ to overcome the gap between the empirical models. The probabilistic models considered penetration rate prediction and rock mass relationship between the TBM. However, it is important to note that these statistical models frequently rest upon presuppositions or approximations concerning the interplay between two variables, leveraging distinct mathematical functions to delineate their relational dynamics. Their capacity to comprehensively encapsulate intricate and non-linear circumstances might sometimes be insufficiently robust, particularly when confronted with outliers or instances of extraordinary data. On the other hand, numerous researchers^4,19–27^ have used artificial intelligence models to predict TBM performance, penetration rate, torque and thrust, cutter wearing, and lithology identification using geological information and TBM operational parameters. Despite integrating TBM parameters and lithology data into prior intelligent models, most research has focused on traditional methods for predicting how the conditions of the ground affect the operation of TBMs. These studies also look at how different operating factors of TBMs are related to each other. Apart from TBM performance prediction approaches, there are a few TBM-logged data-based studies: rock mass characterization^28^ and machine–soil interaction^29^. Although there is limited research exploring the direct relationship between TBM-logged data and operator decisions during excavation to mitigate human error in TBM monitoring, our investigation highlights a noteworthy gap in practical applications that our study seeks to address. Furthermore, a significant challenge in many of these efforts is the requirement for manually adjusting hyperparameters or using basic tuning methods like random search, grid search, and genetic algorithms. However, the widely used hyperparameter tuning algorithms hinder their integration to the industrial scale. Additionally, they are model-based approaches unsuitable for evaluating different models and providing optimum hyperparameters simultaneously^30^. Nevertheless, the Optuna approach is based on Bayesian optimization and can provide runtime issues and a robust production environment and performance^31^.

Concerning the previous comprehensive literature review, this study proposes the following novel contributions:

- The primary contribution of this research is the achievement of synergy between operator decisions, representing TBM parameters observed through the operator's experience, and TBM logs generated during machine-ground interactions without human intervention and recorded by the TBM monitoring system. This TBM-logged data is directly influenced by the actions of the TBM operator, and human error constitutes a significant factor in the reduction of excavation efficiency. The proposed intelligent system is designed to establish a robust correlation between human decisions and machine responses, enabling the monitoring of TBM jack speed and torque under optimal conditions. This system effectively prevents excessive and reduced tunnelling speeds, thus enhancing the overall performance of the TBM while mitigating human-related errors.
- Data synchronization between a large TBM logged data (torque and jack speed) and a small TBM operator dataset (which are manually set by the TBM operators). It combines data-driven machine-learning techniques with real-time operator input to optimize TBM performance. This approach can potentially enhance efficiency, reduce downtime, and improve the quality of tunnel excavation.
- Supervised regression models have been integrated with the Optuna; automatic hyperparameter tuning. By automating the fine-tuning of hyperparameters, these models enhance the adaptability and efficacy of intelligent systems, propelling them toward unprecedented levels of performance and responsiveness.

This research conducts a thorough study of how operators and machines work together in tunnelling. It works on improving prediction methods, making the fine-tuning of settings automatic, and getting the best performance out of the machines. The goal is to make tunnelling more accurate and efficient and to create new ways to combine human skills and artificial intelligence in tunnelling projects.

## Data

### Project description

**Figure 1:**
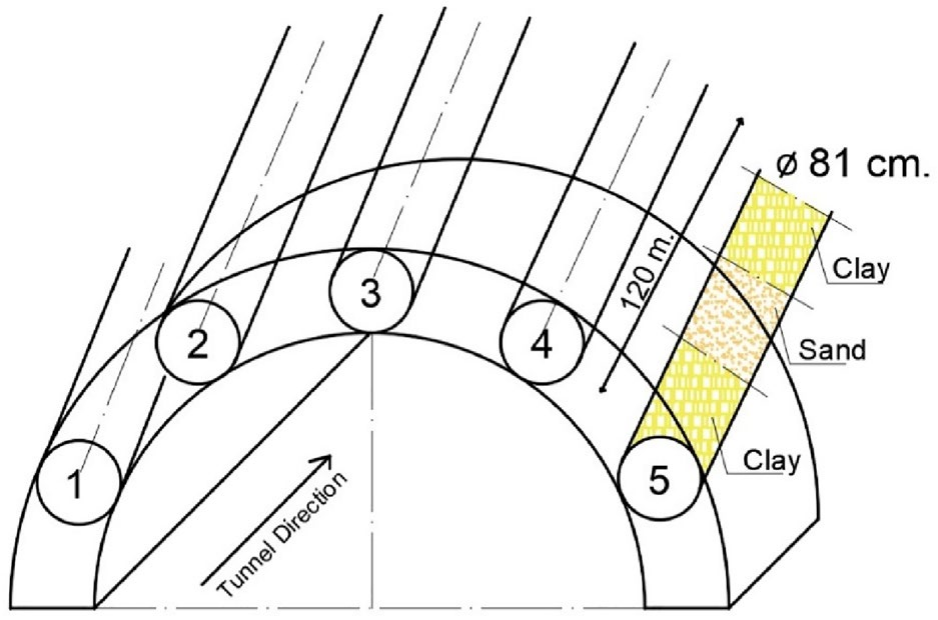
Illustration showcasing the umbrella support design and excavation process. Circular shapes indicate pipe holes (from number 1 to 5), and the cross-sectional view of pipes highlights excavated holes created by the MSTBM.

The ongoing tunnelling project involves the construction of a highway bypass tunnel within Japan. The primary tunnel is being excavated through conventional drilling and blasting techniques. Given the soft ground conditions at the entrance of the tunnel project, a method involving umbrella pipe support excavation is implemented to enhance tunnel roof stability. Figure 1 illustrates the execution of the excavation procedure for umbrella pipe support by utilizing the micro pipe jacking slurry tunnel boring machine (MSTBM).

The geological composition of the pipes includes sandy clay, sand, brown clay, blue clay, and sandy clay, arranged in sequential order. Due to the resemblances observed in the soil samples, they are categorized as TUC, TUS, and TC, following the details provided in the site investigation report. A summary of the mechanical characteristics of the soft ground is presented in Table 1.

**Table 1:** Mechanical properties of pipe geology.

Soil labels and properties		Elastic module (kN/m^2^)	Cohesion (kN/m^2^)	Friction angle (angle)	Unit weight volume (kN/m^3^)
TUC	Sandy/clay	23,800	110	20	18
TUS	Clay zone	14,500	38	37	17
TS	Sandy/clay	30,800	120	12	19

### Micro slurry TBM machine specifications

**Figure 2:**
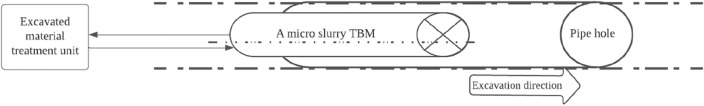
The fundamental idea behind the MSTBM's operation centres on tunnel excavation and transporting excavated material via pipelines. The excavated material undergoes soil treatment and pressure control at the cutter face to improve stability.

An umbrella pipe support excavation was carried out utilizing the micro tunnelling technique for a Japanese highway bypass tunnel project. Figure 2 demonstrates a concise overview of the operational concept of the MSTBM, while Table 2 outlines the detailed specifications of the MSTBM.

**Table 2:** MSTBM specifications.

Machine specifications	
Shield outer diameter (cm)	83
Shield length (m)	5.2
Thrust force (kN/m^2^)	245
Torque (kN·m)	15.2

## Data preprocessing and synchronization

The established procedure of regulating the driving parameters of the MSTBM is commonly applied for construction objectives, frequently involving the utilization of temporal averaging on the collected data. This research investigates a connection between the MSTBM operator's decision-making data (input), and MSTBM logged data (output). The tunnel roof is equipped with 24 pipe holes, each containing 18 pipes. The dataset was collected from the MSTBM using TBM-logged data acquisition at 1-s intervals and the TBM operator's monitoring decisions for each 250 mm digging stroke. The TBM-logged data contained 678,325 data points, while the operator data contained 386. The dataset was synchronized into a single data frame due to discrepancies between the operator and TBM-logged data. After synchronizing the data (309 data points; the number of synchronized data) for the AI model, the dataset was divided into 80% training data (247 data points) and 20% testing data (62 data points) for model validation. In our focused approach to understanding TBM propulsion parameters, the model initially employed the operator's jack speed input to predict the TBM-logged jack speed, establishing a direct correlation between operator decisions and the machine's performance. Subsequently, leveraging insights from the first prediction, the model aims to predict the TBM-logged torque, using operator inputs related to torque settings. This sequential predictive modelling emphasizes the pivotal role of operator decisions in influencing the main propulsion parameters of the TBM, namely jack speed and torque. Table 3 shows a descriptive analysis of operator torque/jack speed and TBM-log torque/jack speed values. Table 4 illustrates the input and output parameters of the model. The operator’s jack speed and torque were input, and TBM-log’s torque and jack speed were output of the model separately.

**Table 3:** Descriptive analysis of the operator-set torque/jack speed and TBM-log torque/jack speed.

Descriptive analysis of operator-set values and TBM-log data				
	Operator-set torque	TBM-log torque	Operator-set jack speed	TBM-log jack speed
Mean	3.22	3.11	86.62	85.25
Median	2.97	2.85	87	81.25
Std	1.88	1.85	58	58.08
Min	0	0	0	0.47
Max	10.58	7.87	278	180

**Table 4:** Predictive model’s input and output.

Input parameters—operator-set	Output parameters—TBM log
Jack speed	Jack speed
Input	Output
Torque	Torque

The data acquisition process is detailed in sections “TBM logged data (target)” and “TBM operator data (input)”, respectively. Each section also includes a description of the data preprocessing steps.

### TBM logged data (target)

The TBM-logged records comprehensive details about the TBM's operational condition and the progression of construction activities^29^. This dataset is categorized into several groups, encompassing aspects like positioning, hydraulic pressures, management of fluids, status indicators, electrical currents, voltages, thrust force, passive lateral earth pressure, sludge flow, jack stroke, jack speed, and excavation speed. This paper will focus on MSTBM torque and jack speed due to the main machine's advancement to the tunnel face. This selection aims to elucidate how intricate torque and jack speed control influence MSTBM operations. The MSTBM-logged data acquisition is continuous for each 1 s for one pipe in the excavation stroke. Therefore, certain pipes in the MSTBM-logged data exhibit numerous data points.

Given the unique characteristics of the dataset, the TBM-logged data was extracted with the following steps: extracting TBM data for a specific ring number and its corresponding operator stroke, filtering the TBM data only to include strokes for which there are corresponding operator data, calculating the mean values of the TBM data for each unique combination of ring number and operator stroke. This statistical analysis provides insights into the average TBM performance for different conditions. Equation (1) presents extraction of the each unique value.

1 $$\left({s}_{t},{p}_{t}\right)= {f}_{1}\left({s}_{o},{p}_{o}\right)$$

where ${s}_{o}$ represents the operator's excavation stroke, ${p}_{o}$ represents the corresponding pipe number in a pipe hole. For MSTBM logged data, ${s}_{t}$ represents the excavation stroke and ${p}_{t}$ refers to the pipe number in the pipe hole. Equation (2) indicates stroke-based data aggregation, briefly.

2 $${D}_{agg}= {f}_{2}({D}_{t},{s}_{o})$$

where ${D}_{t}$ represents the MSTBM-logged data, and ${D}_{agg}$ denote the aggregated data for each operator's stroke, ${f}_{2}$ involves calculating the mean values of ${D}_{t}$ with the excavation stroke ${s}_{o}$.

**Figure 3:**
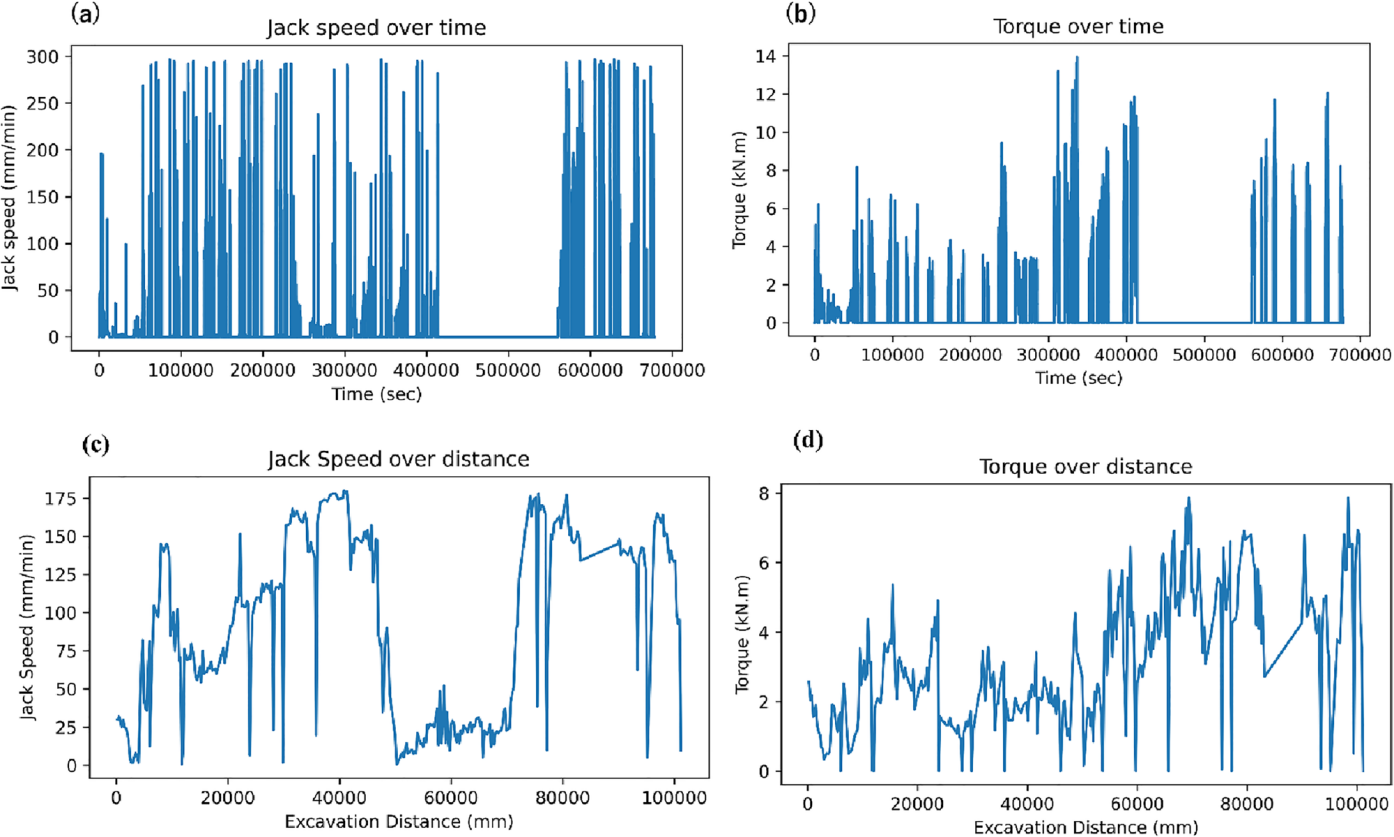
(**a**) Raw TBM-log jack speed in the time domain, (**b**) raw TBM-log torque in the time domain, (**c**) extracted jack speed over distance, and (**d**) extracted torque over distance.

Additionally, it's worth noting that plotting data based on either time or distance frequently yields distinct perspectives on the dominant mechanisms at play. As such, distance-based filtering has been extensively employed in analytical procedures. The logged torque and jack speed were extracted from the time domain to distance-based with plotting versus excavation distance to observe logged behaviour. Figure 3a illustrates the logged jack speed, Fig. 3b shows logged torque distribution based on time interval, Fig. 3c presents the extracted logged jack speed over distance, and Fig. 3d expresses the extracted logged torque over distance.

### TBM operator data (input)

The operator-set torque and jack speed data acquisition differ from the MSTBM-logged data, which is not a time-domain dataset. Operator data, specifically referring to the parameters of torque and jack speed set by the TBM operators, was recorded systematically, though the method of recording may differ slightly from that of automatically logged TBM data. Operator data, specifically parameters such as torque and jack speed set by the TBM operators, is recorded manually through the control system interface in the TBM’s main control room. Operators input these values based on their assessments and the excavation requirements at any given time. This manual input process results in a dataset that reflects the operators’ decisions and intended machine settings. The dataset recorded operational parameters corresponding to each 250 mm stroke of the machine. The operator dataset was 386 data points and synchronized, extracting the operator’s torque and jack speed to include the strokes for which there is associated TBM data. This step ensures that operator data is consistent with the available TBM data.

**Figure 4:**
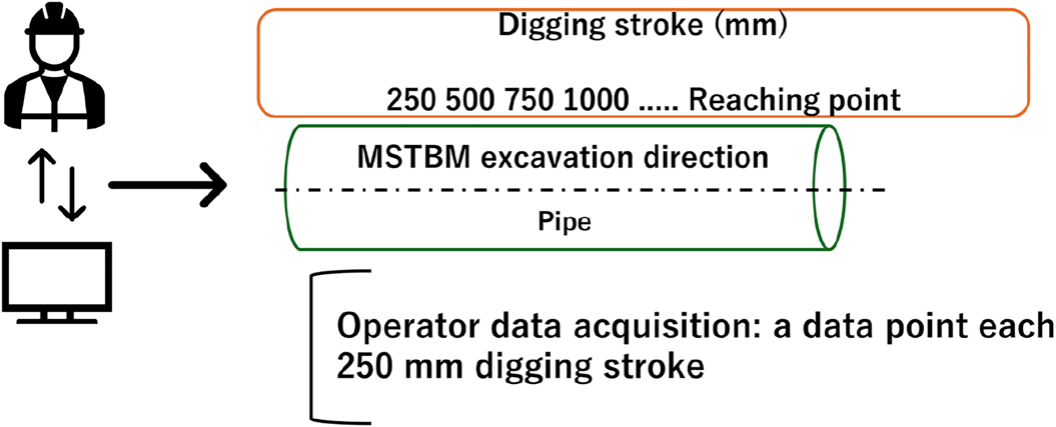
TBM operator data acquisition is based on each 250 mm digging stroke. Each operational data point corresponds to each 250 mm digging stroke.

Figure 4 demonstrates a representation of jack stroke-based data acquisition.

**Figure 5:**
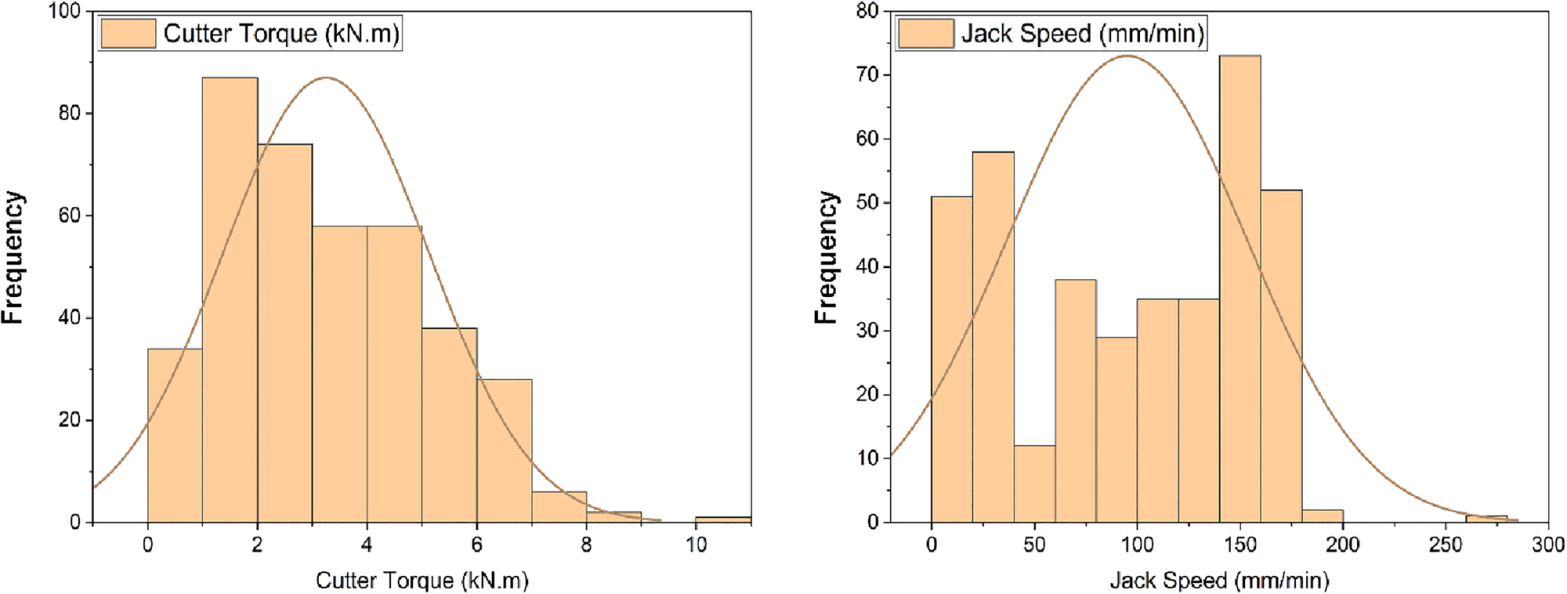
The micro slurry pipe jacking TBM's jack speed and torque frequency distribution histogram. The y-axis refers to the frequency, and the x-axis corresponds to the values of the parameters.

Figure 5 depicts the frequency distribution of the data for operator-monitored torque and jack speed. Owing to the discernible distribution pattern observed in the frequency plot, the normalization and feature conversion technique is utilized to adapt the data into the range of [0–1]. This approach aims to reduce the influence of data magnitude on variability, as discussed by^4^. The process of min–max normalization is defined by Eq. (3), as introduced by^32^.

3 $${x}_{scaled }= \frac{x-{x}_{min}}{{x}_{max}-{x}_{min}},$$

where ${x}_{scaled}$ is the normalized data, x is the raw data, and ${x}_{max}$ and ${x}_{min}$ are the maximum and minimum values of the dataset, respectively.

## Methodology

Following data preprocessing, the dataset is primed for utilization with the Optuna-assisted AI models^31^. Optuna applied a 1000-times tuning trial with several hyperparameters to increase R^2^. The RF model was selected as the best regressor among DT, kNN, XGBoost, SVM, and ANN regressors. The 1000-time trials allowed Optuna to provide the selected model with its hyperparameters, R^2^, MSE, and MAE. The XGBoost and the ANN were trained out of DT, kNN, and RF objective function due to XGBoost and the ANN hyperparameters and architecture were different from traditional machine learning models. Table 5 illustrates the model comparison after the Optuna integration for the prediction of jack speed and torque.

**Table 5:** Comparison of the regression models and computation time.

Model name	R^2^ (%)	MSE	MAE	Computation time (s)
Jack speed prediction				
Random forest	96	119.7	4.42	0.35
Decision Tree	96	124.5	4.67	0.56
k-NN	95	130.62	5.07	0.36
XGBoost	95	137.4	5.67	0.42
ANN	92	118	4.43	3.43
SVM	71.25	118.25	4.43	23.19
Torque prediction				
Random forest	83	0.62	0.42	0.35
Decision Tree	82	0.60	0.40	0.58
k-NN	81	0.63	0.43	0.39
XGBoost	80	0.69	0.48	0.41
ANN	92	0.81	0.75	3.57
SVM	61.25	0.85	0.73	25.26

**Figure 6:**
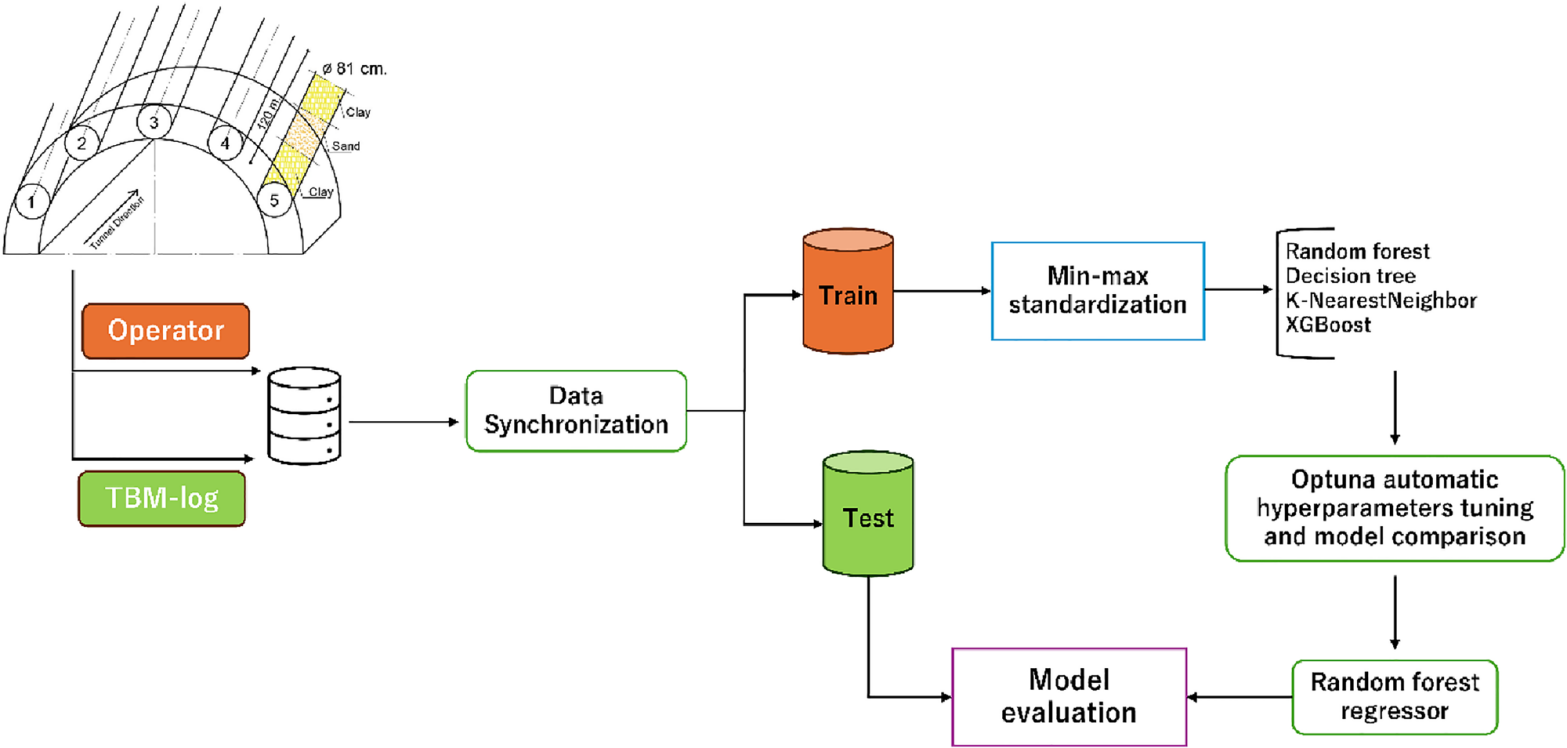
The Optuna-assisted AI model structure. It can be seen how to synchronize TBM-logged and operator data as a single data frame for the best regressor model with Optuna.

As a result, section “Optuna-assisted AI model” delineates the machine learning model's elucidated through employing Optuna. section “Selected model structure and hyperparameters” depicts Optuna-decided hyperparameters and features the importance of hyperparameters and the relationship between hyperparameters for deciding the best model. Furthermore, section “Evaluation metrics” clarifies the evaluation metrics used, which encompass the correlation coefficient (R^2^), mean squared error (MSE), and mean absolute error (MAE). Figure 6 indicates the Optuna-assisted prediction model briefly.

### Optuna-assisted AI model

Akiba et al.^31^ expressed that Optuna is defined as a define-by-run API. Optuna provides a dynamic space for the user to build the parameters, and efficiently incorporating both search and pruning methods and a straightforward setup process results in a versatile architecture suitable for various applications. These purposes encompass scalable distributed computing and lightweight experiments conducted through interactive interfaces. The concept of the “define-by-run” API becomes more explicit when examining actual code. Optuna approaches hyperparameter optimization as a procedure that minimizes or maximizes an objective function. The objective of using Optuna for regression tasks is to maximize this R^2^ score. This function takes a set of hyperparameters as input and yields a validation score as its output. In our research, the objective function is to increase R^2^. Equation (4) explains the Optuna R^2^ score maximization function.

4 $$\begin{aligned} &{\theta }^{*}=argmax{R}^{2}\left(\theta \right)\\ &\theta \in D \end{aligned}$$

where ${R}^{2}\left(\theta \right)$ denoted the ${R}^{2}$ score of the model trained with the hyperparameters vector $\theta$, D is the domain for each hyperparameter.

**Figure 7:**
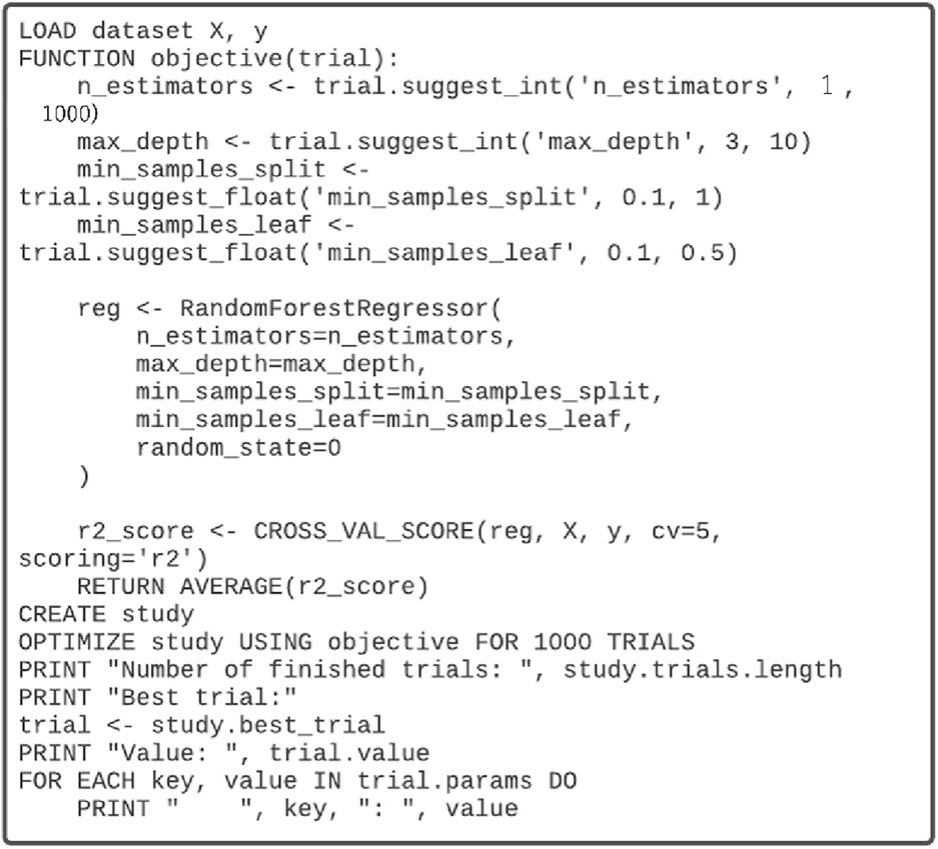
Optuna was employed to optimize hyperparameters for Random Forest (RF), Decision Tree (DT), k-Nearest Neighbors (kNN), SVM, XGBoost, and ANN regression models collectively. By conducting 1000 trials, the best model was selected based on the R^2^ score, facilitating comprehensive model comparisons and the identification of optimal machine learning models with precise hyperparameter combinations for optimal dataset performance.

On the other hand, Liashchynskyi and Liashchynskyi^33^ compared three widely used hyperparameter tuning methods—random search, grid search, and the genetic algorithm—highlighting their limitations in the context of industrial-scale applications. The drawbacks include the inefficiency of grid search in high-dimensional spaces, the lack of systematic exploration in random search, and the genetic algorithm’s dependency on parameter fine-tuning. In contrast, Optuna offers a more efficient and scalable solution, leveraging a Bayesian optimization strategy with a pruning mechanism, a user-friendly interface, and the capability for both single and multi-objective optimization. These advantages make Optuna a superior choice for hyperparameter tuning in complex and large-scale machine-learning tasks. Figure 7 demonstrates how to work the objective function of the Optuna for the RF regression model representatively.

### Selected model structure and hyperparameters

**Figure 8:**
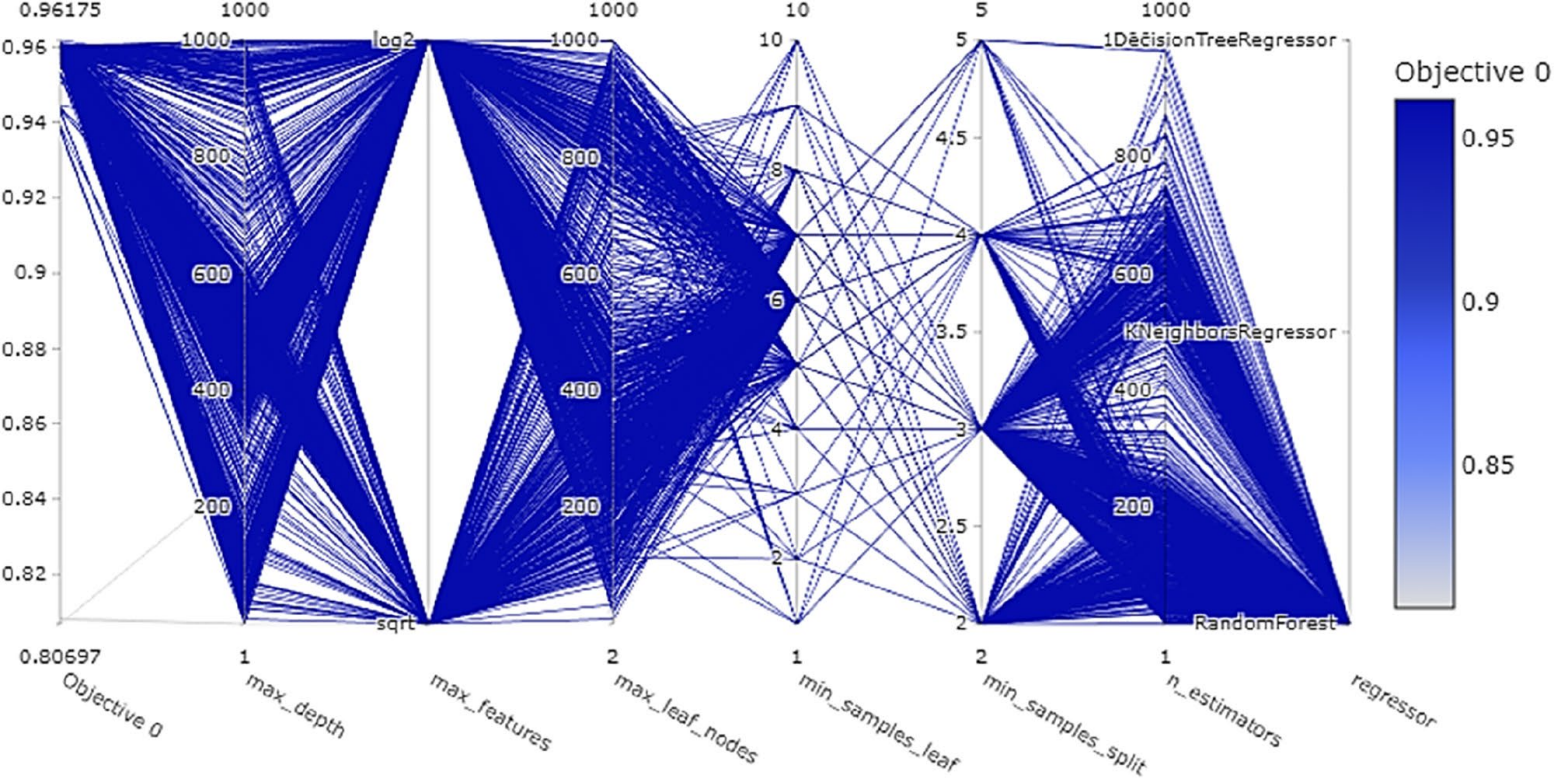
The Optuna parallel coordinates plot reveals the interplay between high-dimensional hyperparameters and machine-learning models following 1000 trials. It visualizes the optimal hyperparameter configurations for regression models, enhancing the R^2^ score thereby aiding model selection.

Following the application of Optuna, the best model was decided as a random forest regressor to predict the jack speed and torque. Optuna compared kNN, DT, RF, and SVM models while optimizing hyperparameters for the best model. Owing to the different requirements for the XGBoost and ANN, the XGBoost and ANN were trained separately from the kNN, DT, RF, and SVM. Figure 8 illustrates the parallel coordinate plot that shows the relationship between high-dimensional parameters. It provides parallel processing and optimization during multiple trials among numerous ranges of parameters with several models simultaneously.

It's important to note that the trials for kNN and DT regressors were included in the optimization process; however, due to the scale and density of the trials for the RF regressor, they might not be readily discernible in the plot. The kNN and DT regressors had a different set of hyperparameters compared to the RF regressor, and the visualization adapts to illustrate these when relevant trials are selected or highlighted. In cases where the RF regressor dominated the plot due to a larger number of trials or wider hyperparameter ranges, the trials for kNN and DT might be overshadowed or less represented in the visualization. This exploration is crucial for understanding the dynamic interplay between various hyperparameters and the model's performance. When focusing on Fig. 8, a pivotal observation comes to light. Specific hyperparameters are keystones in the model's architecture, significantly shaping its predictive prowess. In particular, it becomes apparent that one hyperparameter, denoted as max depth, emerges as indispensable, exhibiting an intrinsic influence surpassing the model. This comparative analysis underscores the nuanced hierarchy of hyperparameters. Table 6 identifies the selected hyperparameters for the RF model.

**Table 6:** Random forest model hyperparameters and values.

Hyperparameters	Values
Criterion	Absolute error
Max depth	953
Max feature	Log2
Max leaf nodes	249
Number of estimators	4
Min samples split	3
Min samples leaf	6
MSE	119.7
MAE	4.47
R^2^ score	96%

### Evaluation metrics

The performance of the explainable neural networks model was assessed using three evaluation metrics: R^2^, MSE, and MAE. Equation (5) presents the formulation for R^2^, which quantifies the extent to which the model's inputs explain the variability in the dependent variable^34^.

5 $${R}^{2}=1-\frac{{\sum }_{i=1}^{n}{({\widehat{y}}_{i}-{y}_{i})}^{2}}{{\sum }_{i=1}^{n}{\left({\overline{y} }_{i}-{y}_{i}\right)}^{2}}$$

where ${\widehat{y}}_{i}$ represents the estimated value of the data, ${y}_{i}$ is the actual value, ${\overline{y} }_{i}$ is the mean of the predicted value, and *n* is the total dataset number.

The Mean Squared Error (MSE) proves effective when identifying outliers is important. The L2 norm is utilized, assigning greater significance to outliers. Specifically, when the model generates an inferior prediction, the error amplification occurs due to the squaring mechanism within the equation. Equation (6) defines the MSE equation^34^.

6 $$MSE=\frac{1}{m}\sum_{i=1}^{m}{\left({X}_{i}-{Y}_{i}\right)}^{2}$$

where ${X}_{i}$ is the predicted and ${Y}_{i}$ is the actual value.

The Mean Absolute Error (MAE) is more suitable when outliers indicate anomalies within the dataset. Unlike MSE, MAE doesn’t heavily penalize outliers during training, resulting in a comprehensive and balanced performance evaluation for the model. Conversely, if the test set contains numerous outliers, the model's performance will be moderately affected. Equation (7) outlines the formulation of MAE.

7 $$MAE=\frac{1}{m}\sum_{i=1}^{m}\left|{X}_{i}-{Y}_{i}\right|$$

where ${X}_{i}$ is the predicted and ${Y}_{i}$ is the actual value.

In summary, these evaluation metrics—R^2^, MSE, and MAE—provide a comprehensive understanding of the Optuna-assisted AI model's performance, catering to various scenarios involving data variability and outlier presence within the dataset.

## Results

**Figure 9:**
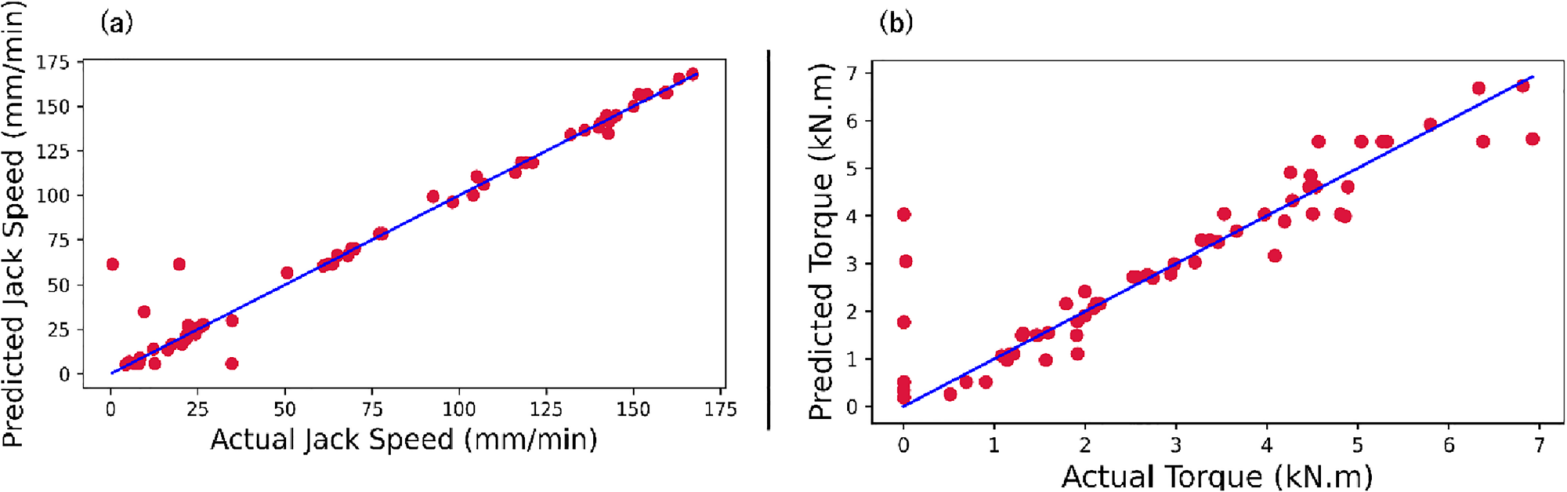
(**a**) Predicted and actual data distribution of the jack speed. (**b**) Predicted and actual data distribution of the torque. The red circles refer to the data points, and the blue corresponds to the best-fit line.

The random forest regressor's performance was assessed through R^2^, MSE, and MAE metrics. The RF model demonstrated an R^2^ value of 96%, with MSE and MAE scores of 119.7 and 4.47 for the prediction of the jack speed, respectively. The model provided R^2^ values of 83%, MSE 0.62, and MAE 0.42 for predicting the torque, respectively. The model's predictions were visually represented using graphs depicting predicted outcomes alongside actual results. Figure 9a compares the RF model's predictive performance of jack speed, and the actual data points, and Fig. 9b shows the RF model's predicted and actual torque. Most data points cluster around the line of best fit, underscoring the model's proficiency in accurately forecasting jack speed values and torque. However, it can be seen that there were instances where the actual torque recorded was zero or close to zero. Such occurrences might arise during periods when the TBM was operational but not actively excavating, such as during setup, maintenance, or other non-cutting stages of the tunnelling process. The presence of these data points was expected and reflects the operational reality of TBM usage, where torque requirements can vary widely based on the stage of operation and the geological conditions encountered.

**Figure 10:**
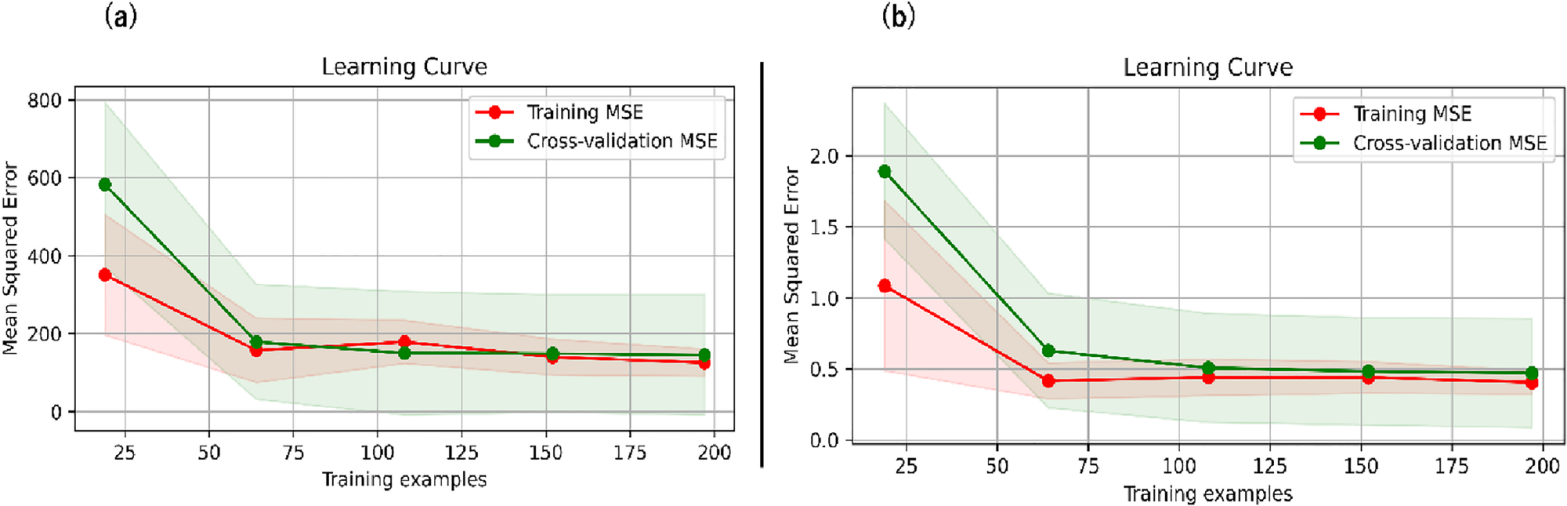
(**a**) Learning curve of the jack speed. (**b**) Learning curve of the torque. The red line refers to the training curve with an MSE of 119.7, and the green line corresponds to the validation curve with an MSE of 0.62.

Nevertheless, scattered data points indicate instances where the model deviates from precise predictions, indicating potential areas for refinement and enhancement. Figure 10a shows an analysis of the RF model's performance through the learning curve for jack speed, and Fig. 10b indicates the model's learning curve of torque. The model's learning curve encompasses training and validation errors evaluated using the MSE metric across varying training iterations.

As depicted in Fig. 10a,b, the model showcased a gradual reduction in training and validation errors, eventually reaching a point of stabilization. This pattern signifies the model's effective learning process and capability to generalize effectively. Notably, the absence of a discernible gap between the training and validation curves indicates that the model was provided with sufficient training data, thereby mitigating concerns about overfitting. Furthermore, the divergence in MSE values—180 for training and cross-validation versus 120 for testing—may initially appear counterintuitive as models typically perform better on training data due to their familiarity with it. The model may generalize better than it memorizes the training data. During the training phase, a robust model learns the underlying patterns without overfitting the noise within the training dataset.

**Figure 11:**
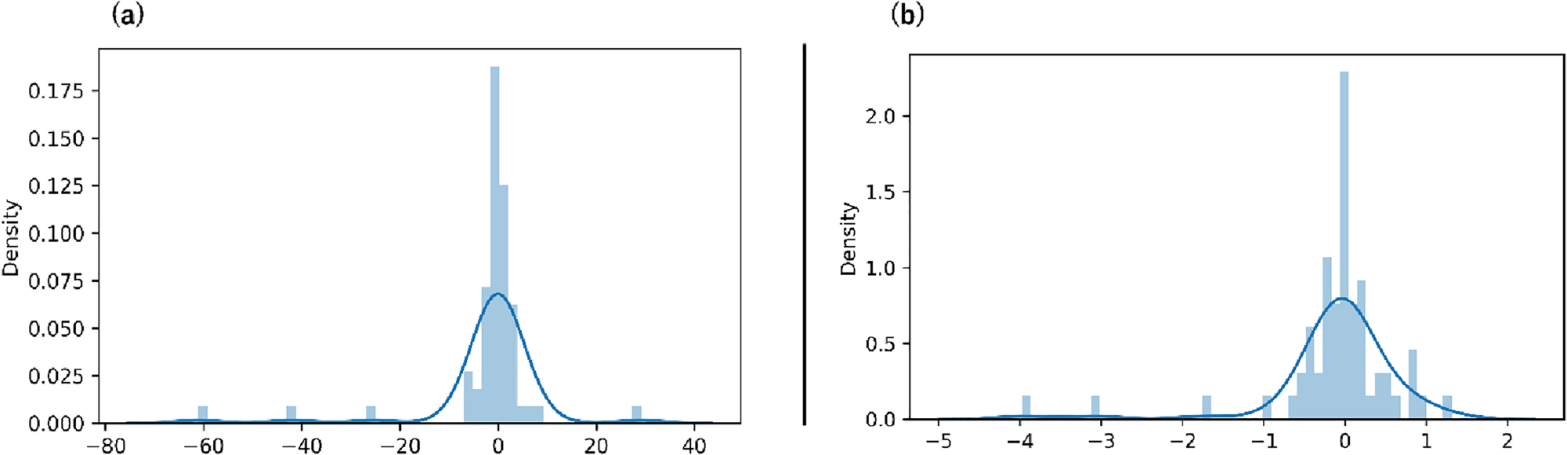
The RF model’s results with (**a**) prediction error histogram distribution of the jack speed and (**b**) torque prediction error histogram.

Additionally, Fig. 11a,b demonstrate that model prediction errors were evaluated using a prediction error histogram for jack speed and torque, respectively. Figure 11a,b depict the distribution of error ranges, with the x-axis denoting the magnitude of errors and the y-axis representing the frequency and density of occurrences. An adeptly performing model would showcase a symmetrical distribution tightly clustered around zero error, signifying minimal prediction disparities. Figure 11a,b reveal a prominent central peak around zero error, underscoring the model's propensity to make accurate predictions.

## Discussion

This research has yielded valuable insights by exploring the connection between an MSTBM operator's decisions and the data collected from the MSTBM during MSTBM operations within umbrella support excavations. Among a range of operational parameters governing MSTBM advancement, the study specifically focused on jack speed and torque. The obtained results highlight the robust predictive capabilities of the RF model in monitoring construction torque and jack speed. This model exhibits a superior ability to anticipate these vital parameters accurately. Consequently, there exists a compelling opportunity to employ this well-trained RF model to optimize MSTBM parameters, effectively managing the advancement of the machine towards the tunnelling face. In light of these findings, it can be contended that the Optuna-assisted AI model is characterized by a high degree of generalizability. This transition from human-based monitoring to an AI-driven approach holds promise for more efficient and precise MSTBM operations. The study's outcomes thus pave the way for potential advancements in autonomous tunnel boring. It can be noted that the model was going to be evaluated using one different pipe hole dataset using a pre-trained model; however, owing to the redundant failure of the MSTBM-control panel, the MSTBM-logged data from the other pipe hole was not recorded. Therefore, the Optuna-assisted RF model could be examined with a different dataset to see its performance for real-life applications in further research.

## Practical applications

The practical implementation of our AI model in the tunnelling sector is an advancement born from the extensive knowledge gained during this research. The model, enhanced by Optuna for industrial-scale deployment, embarks on a predictive and adaptive phase post rigorous training. As the operator maneuvers the TBM through the excavation process, the model diligently processes real-time operational data and MSTBM-logged information, alongside the operator’s manual adjustments to jack speed and torque. It employs this data to prognosticate the optimal parameters that suit the immediate tunnelling environment.

The utility of the model shines when actual conditions deviate from the anticipated course. It triggers a feedback loop to the operator, presenting a detailed analysis of why certain adjustments may prove beneficial. This level of interactivity fosters a seamless marriage of human judgment and AI-generated foresight, thus streamlining the fine-tuning of operational parameters to boost efficiency and precision.

**Figure 12:**
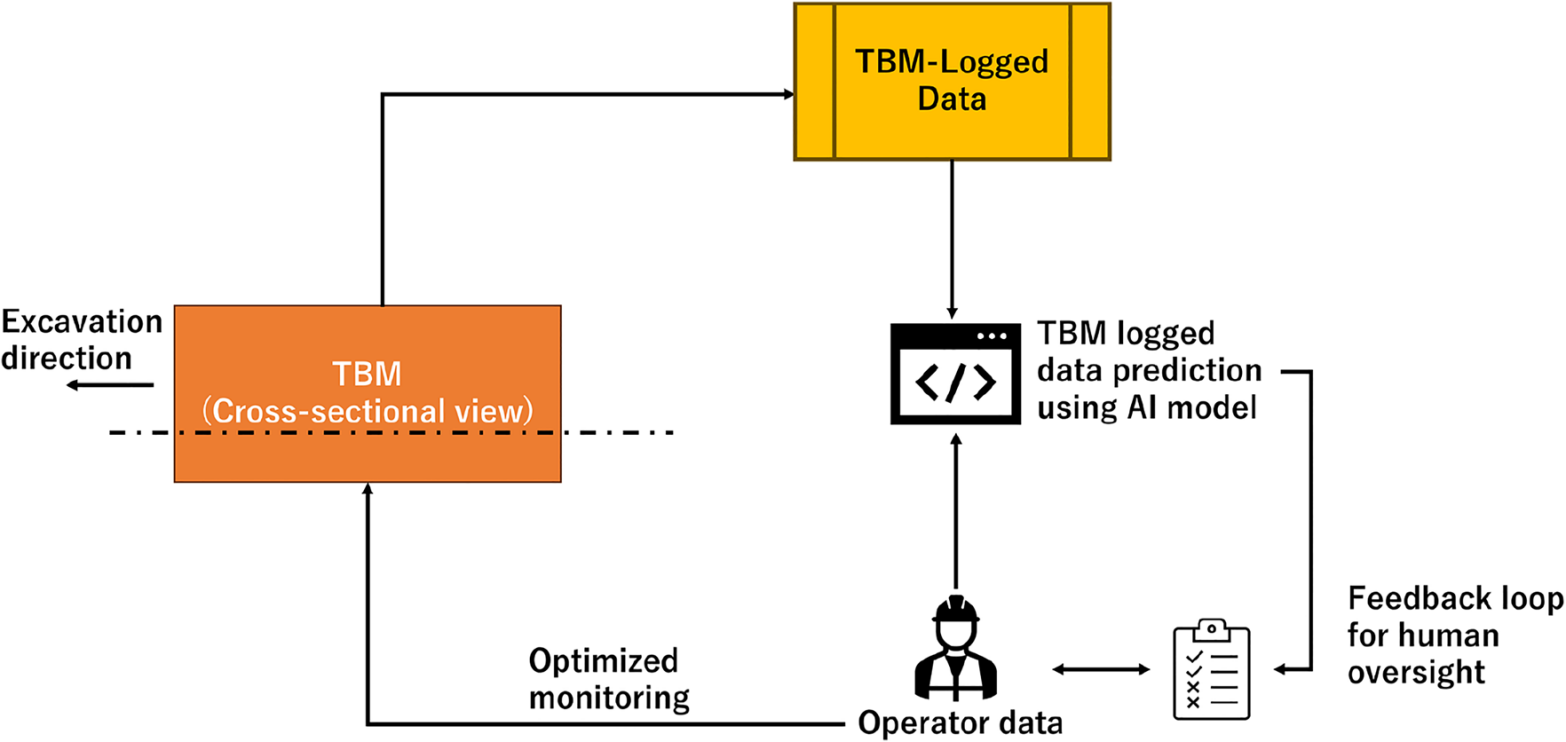
A TBM monitoring system that combines AI with TBM-logged data and operator decisions for enhanced monitoring. The AI-assisted model predicts TBM-logged data based on the operator input. The operator can see predicted jack speed and torque with a feedback loop to optimize TBM monitoring while avoiding human error.

Moving beyond collaboration, the model is designed to autonomously recalibrate parameters, drawing on real-time data feeds. In this autonomous mode, while the AI assumes direct control over adjustments, it simultaneously equips the operator with a transparent understanding of its decision-making process. This dual approach not only empowers the operator with a robust decision-support tool but also ensures that the AI remains focused on enhancing the TBM's operational performance. The AI's dual capacity for both suggestion and autonomous adaptation is encapsulated in Fig. 12, which outlines the AI-assisted TBM monitoring's practical application. The model stands as a beacon of innovation, guiding parameter decisions with precision, backed by predictive analytics and operator expertise.

The current research signifies a pivotal step towards a new era in MSTBM operations. This study paves the way for enhanced efficiency, optimized excavation, and sustainable tunnelling practices. The potential synergy between AI's computational prowess and human expertise holds the promise of reshaping the tunnelling landscape for the better. As AI advances, realizing AI-assisted MSTBM control seems attainable and transformative.

Notwithstanding its advantages, the Optuna-assisted AI model does have certain limitations. These constraints can be outlined as follows:

1. The model performance might vary when dealing with geological conditions not well-represented in the training data. The model's predictive accuracy might be compromised in scenarios with significant geological variations. This constraint is the black box of the model.
2. Optuna's objective function shows different computation times for machine learning models and number trials.
3. The AI model's complex algorithms can pose challenges in terms of interpretability. Understanding the rationale behind its decisions might prove difficult for operators and engineers, potentially impacting their trust in the model's recommendations.
4. Environmental conditions, such as ground stability changes or unexpected geological features, can significantly affect TBM operations. The AI model might struggle to adapt to abrupt changes not encountered during training.
5. The AI model learns from historical operator decisions, which might not always represent a single best approach. Operator preferences can vary, and the model might not capture every nuanced decision-making pattern. The model was constructed using operational parameters from micro-tunnelling TBMs, implying that utilizing operational parameters from larger TBMs or those designed for hard rock conditions would yield divergent outcomes.
6. Due to the data synchronization between extensive and small datasets, complex preprocessing can be figured out in future studies.
7. Automating certain aspects of TBM control raises ethical questions regarding the balance between human expertise and machine decision-making. Ensuring responsible and transparent use of AI in critical infrastructure projects is essential.

## Conclusions

In conclusion, this research presents a significant advancement in tunnel boring operations by leveraging the power of artificial intelligence. Through the development and exploration of an Optuna-assisted AI model, the study has shed light on the intricate dynamics between MSTBM operators' decisions, MSTBM-logged data, and the control of crucial parameters like jack speed and torque. The findings underscore the transformative potential of AI in optimizing MSTBM operations and enhancing efficiency across diverse geological and operational contexts. The insights gained from this study hold practical implications for the tunnelling industry, paving the way for a future where AI-driven systems collaborate seamlessly with human expertise. In particular, the successful performance of the RF model in monitoring construction torque and jack speed signals the feasibility of implementing AI models to fine-tune MSTBM parameters using Optuna.

The main conclusions of this research are:

1. Unlike typical prior investigations focusing on mechanized tunnelling in hard rock conditions, this study specifically dealt with parameters of a micro-slurry MSTBM machine operating in soft ground environments.
2. The research highlights the predictive capabilities of the RF model. Its evaluation metrics are R^2^ of 96%, MSE of 119.7, and MAE of 4.47 for jack speed, and R^2^ of 83%, MSE of 0.62, and MAE of 0.42 for torque prediction. While reasonable results were obtained for predicting jack speed, the model's performance was comparatively weaker in predicting torque. However, torque's prediction error and learning curve scores were lower than the predicted jack speed.
3. The research provides a new insight into the relationship between the MSTBM operator and MSTBM logged data to control the machine advancement.
4. Compared to the previous applications, the study showed Optuna integration to the machine learning model for automatic hyperparameters tuning. This study introduces a pioneering approach by integrating Optuna into the machine learning model for automated hyperparameter tuning. This advancement underscores the potential for optimizing the model's performance through efficient parameter calibration.
5. The research indicated human expertise AI models can handle control MSTBM parameters. Human-expertise models could be used instead of sensor-based control systems in the future. This offers an alternative to sensor-based control systems, with potential benefits including cost-effective data acquisition and streamlined preprocessing.

These main conclusions collectively underline the contributions of this research, offering insights into the dynamics of TBM operations, the potential of AI-assisted control, and the efficacy of innovative model integration. The implications of these findings extend to optimizing MSTBM performance, enhancing operational efficiency, and charting a course toward intelligent and adaptive tunnel boring operations.

## Data Availability

The datasets generated and/or analysed during the current study are not publicly available due to the confidential dataset from the TBM company, and required permission from the company for sharing publicly but are available from the corresponding author upon reasonable request.

## Software support

Python libraries have been used to implement the methodology.
